# Correction to “Small and Stable Pancreatic Cysts Are Reassuring During Surveillance: Results From the PACYFIC Trial”

**DOI:** 10.1002/ueg2.70136

**Published:** 2025-11-01

I. J. M. Levink, et al., “Small and Stable Pancreatic Cysts Are Reassuring During Surveillance: Results From the PACYFIC Trial,” *United European Gastroenterology Journal* (2025).

There were typographical errors in the text under the bar chart correct of Figures 2B and 3B. There were also typographical errors in the captions of Figures 2 and 3. The revised figures are as follows:

Figure 2:


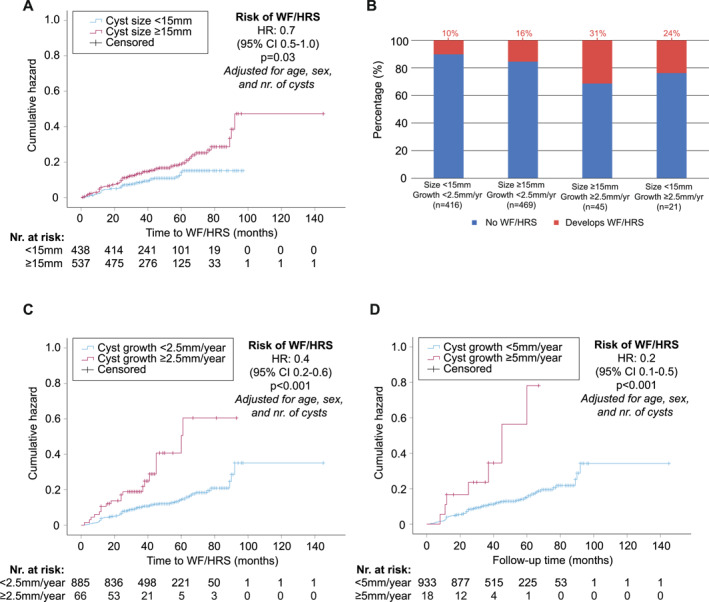


Figure 3:


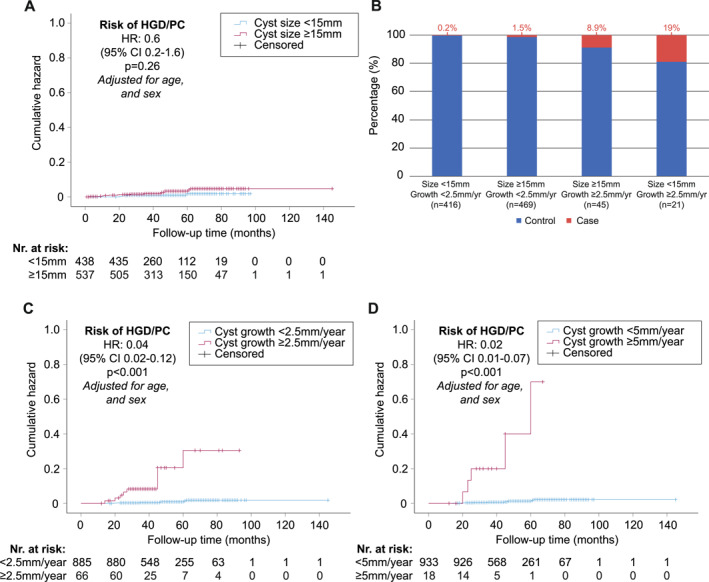


The caption of Figure 2 should have read: “Cox proportional hazard plots showing the risk of developing worrisome features (WFs) or high‐risk stigmata (HRS) (A, C, and D). The hazard ratio (HR) per cyst size (A) and cyst growth group (C, D). (B) The absolute risk per cyst size and growth group.”

The caption of Figure 3 should have read: “Cox proportional hazard plots showing the risk of developing high‐grade dysplasia (HGD) or pancreatic cancer (PC) (A, C, and D). The hazard ratio (HR) per cyst size (A) and cyst growth group (C, D). (B) The absolute risk per cyst size and growth group.”

We apologize for these errors.

